# Risk Factors and Long-Term Prognosis for Chylothorax After Total Cavopulmonary Connection in Children: A Retrospective Study From a Single Center

**DOI:** 10.3389/fped.2021.744019

**Published:** 2021-11-18

**Authors:** Liting Bai, Zhengyi Feng, Ju Zhao, Shengwen Guo, Yuanyuan Tong, Yu Jin, Peiyao Zhang, Peng Gao, Yixuan Li, Jinping Liu

**Affiliations:** Department of Cardiopulmonary Bypass, State Key Laboratory of Cardiovascular Disease, Fuwai Hospital, National Center for Cardiovascular Diseases, Chinese Academy of Medical Sciences and Peking Union Medical College, Beijing, China

**Keywords:** total cavopulmonary connection, chylothorax, risk factors, prognosis, children

## Abstract

**Background:** Chylothorax is a severe complication after total cavopulmonary connection (TCPC) in children. This study was performed to evaluate the incidence, risk factors, and short- and long-term prognosis for chylothorax.

**Methods:** We retrospectively reviewed the electronic records of patients who underwent TCPC between January 2008 and December 2020 in Fuwai Hospital. Patients were divided into two groups based on the occurrence of post-operative chylothorax. Univariate and multivariate analyses were performed to identify risk factors, and long-term survival was estimated by the Kaplan–Meier method.

**Results:** Of 386 patients included in our study, chylothorax occurred in 60 patients (15.5%). Compared with the non-chylothorax group, the prevalence of prolonged intensive care unit (ICU) stay (*p* = 0.000) and post-operative hospital stay (*p* = 0.000) were greater in patients with chylothorax. Post-operative adverse events in terms of infection (*p* = 0.002), ascites (*p* = 0.001), prolonged pleural effusion (*p* = 0.000), and diaphragmatic paralysis (*p* = 0.026) were more frequent in chylothorax patients. The median follow-up duration was 4.0 (2.0, 6.8) years. The chylothorax group had significantly lower survival rates at 1 year (92.4 vs. 99.3%, *p* < 0.001) and 10 years (84.6 vs. 91.6%, *p* < 0.001), respectively. Having a right dominant ventricle [odds ratio (OR) = 2.711, 95% confidence interval (CI) = 1.285–5.721, *p* = 0.009] and a higher peak central venous pressure (CVP) on post-operative day (POD) 0 (OR = 1.116, 95% CI = 1.011–1.233, *p* = 0.030) were the risk factors for the development of chylothorax after TCPC operation.

**Conclusion:** The incidence of chylothorax in patients undergoing TCPC is lower than previously reported but is associated with poor early- and long-term survival. Having a right dominant ventricle and a higher peak CVP on POD 0 are the risk factors for chylothorax after TCPC operation.

## Introduction

As a lifesaving and life-prolonging procedure, total cavopulmonary connection (TCPC) plays an important role in the therapy of patients with cardiac malformation such as single ventricle and tricuspid atresia. It drives the systemic venous blood to enter the pulmonary circulation through an artificial conduit and bypasses the right atrial and ventricle (1). Although with excellent early survival, there are concerns over the complications induced by this special physiological change. Chylothorax is an infrequent but problematic complication after cardiac surgery. Mery et al. (2) analyzed a large multi-institution database and found that the incidence of chylothorax after Fontan surgery is the highest among various cardiac procedures in children. It has been identified that chylothorax is associated with major mortality and increased length of hospital stay, particularly in patients with single ventricle morphology (3, 4).

Lowest temperature, trisomy 21, a higher vasoactive inotropic score on the day of surgery, and use of an assist device were found to be significantly associated with chylothorax after congenital heart surgery (5). However, there are limited studies evaluating the post-operative chylothorax after TCPC operation. A study (6) investigated the predictor for chylothorax in patients who underwent cavopulmonary connection (included superior cavopulmonary connection and Fontan completion), whereas Lo Rito et al. (7) evaluated the post-operative chylothorax in a cohort of exclusively Fontan completion, but did not find any risk factors for chylothorax. The primary purpose of this study was to identify the incidence and risk factors for the development of chylothorax after TCPC operation. We further evaluated the impact of chylothorax on the early- and long-term clinical outcomes.

## Materials and Methods

### Patients Selection and Data Collection

**Figure 1:**
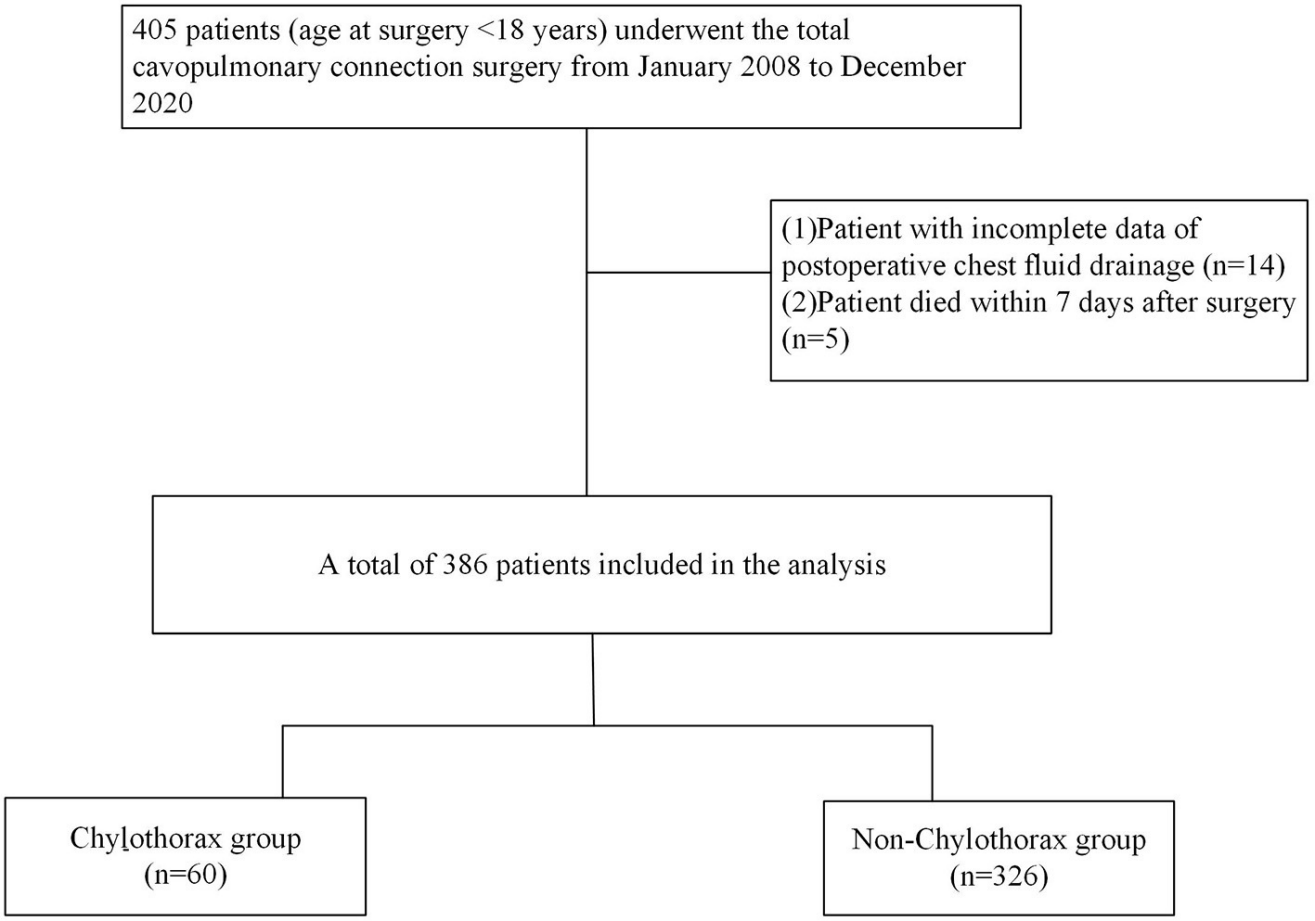
Flowchart of patients.

This single-center retrospective study included all pediatric patients (age at surgery < 18 years) who underwent the TCPC surgery between January 2008 and December 2020 in Fuwai Hospital, Beijing. The hospital's ethics committee approved the study and waived the need for patient consent since the retrospective nature of this study. Patients with missing data of post-operative chest fluid drainage and who died within a week after surgery were excluded. All patients' electronic records in terms of demographic characteristics, cardiac anomalies, echocardiographic parameters, cardiac catheterization hemodynamics, intraoperative data, and post-operative information were reviewed. Based on the occurrence of post-operative chylothorax, patients were divided into two groups: the chylothorax group and the non-chylothorax group (Figure 1).

### Surgical Procedures

After anesthesia, the operation was performed through a midline sternotomy and under parallel cardiopulmonary bypass (CPB) without cardioplegic cardiac arrest unless other concomitant intracardiac procedures were required. The surgery was operated as it was described before (8). The inferior vena cava at the entrance of right atrium and the main pulmonary artery at the root were cut off. The proximal ends of the vessels were closed, and an extracardiac conduit graft was used to connect the distal end of the inferior vena cava and the main pulmonary artery. Intracardiac TCPC was performed for patients with anatomical complexity including apicocaval juxtaposition and separated hepatic vein drainage. A 4- to 5-mm fenestration was made between the right atrial free wall and the conduit. The decision for fenestration was mainly made depending on the surgeon's preference. All patients were admitted to the pediatric intensive care unit (ICU) after TCPC surgery.

### Definition and Outcome Assessment

The diagnosis of chylothorax was confirmed when patients with persistent chest tube drainage ≥5 ml/kg per day beyond post-operative day (POD) 5, and the pleural fluid was chylomicron-positive or drainage with >80% lymphocytes. The Nakata index was calculated as the summation of the left and right pulmonary artery cross-sectional areas indexed to the patient's body surface area. The peak central venous pressure (CVP) of POD 0 was defined as the maximum value of the period since ICU arrival after surgery until 5:00 a.m. on POD 1. The primary outcome of our study was the occurrence of chylothorax. Secondary outcomes were in-hospital death, post-operative complications, mechanical ventilation time, ICU duration, and survival rate after hospital discharge.

### Statistical Analysis

Continuous variables are presented as the mean ± standard deviation or the median with the 25th−75th percentile and compared using the *t*-test or Mann-Whitney *U*-test, respectively. Categorical variables are described in percentages and compared with the χ^2^-test or Fisher exact test. Univariate and multivariate logistic regression models were performed to identify the risk factors for the occurrence of chylothorax. The inclusion criterion for the multivariate logistic regression model was *p* < 0.1 in the univariable analysis. The time-related survival was estimated using the Kaplan–Meier method and compared by the log-rank test. All tests were two-sided, and *p* < 0.05 was considered significant. All statistical analyses in this study were performed using SPSS Statistics 26.0 (IBM, Chicago, IL), GraphPad prism 7.0, and MedCalc 18.2.

## Results

### Perioperative Characteristics of Patients

A total of 386 patients who underwent the TCPC operation were included in our study. Patient characteristics and perioperative data are shown in Table 1. The median age of our population was 4.9 years, median weight was 17 kg, 40.9% were female, and the main primary diagnoses of patients were single ventricle and double outlet right ventricle. All patients underwent CPB, and 29.8% of patients had a fenestration created during the operation. The majority (97.7%, *n* = 377) of the patients underwent an extracardiac tunnel connection. A total of 146 (37.8%) patients had trivial to severe atrioventricular valve regurgitation before TCPC operation. Thirty patients had moderate to severe regurgitation, and 24 of those patients underwent concomitant valve repair during TCPC operation. Concomitant valve intervention for moderate or severe atrioventricular valve regurgitation during the TCPC procedure at our center mainly depends on the preference of the surgeon. All patients underwent CPB with a median time of 143 min, whereas the cardioplegic cardiac arrest was required in 109 patients (28.2%).

**Table 1:** Characteristics of patients with and without chylothorax after TCPC operation.

**Variables**	**Non-chylothora × group**	**Chylothorax group**	***p*-Value**
	**(*n* = 326)**	**(*n* = 60)**	
Age at surgery (years)	4.9 (3.9, 6.6)	4.8 (3.5, 6.2)	0.053
Age ≤ 3.5 years	44 (13.5)	14 (23.3)	0.044
Sex, males, *n* (%)	191 (58.6)	37 (61.7)	0.656
Weight (kg)	17.0 (15.0, 20.0)	16.0 (14.0, 19.0)	0.183
Weight for age *Z* score	−0.44 ± 1.18	−0.51 ± 1.0	0.675
Weight for age *Z* score < -2	22 (7.4)	6 (10.5)	0.595
Hemoglobin (g/L)	176.5 ± 25.0	174.4 ± 28.4	0.563
120–160	94 (28.9)	14 (23.7)	0.414
>160	229 (70.5)	42 (71.2)	0.911
**Cardiac anomalies**, ***n*** **(%)**			
Single ventricle	120 (36.8)	30 (50.0)	0.054
Double outlet right ventricle	89 (27.3)	17 (28.3)	0.869
Tricuspid atresia	64 (19.6)	7 (11.7)	0.143
Pulmonary atresia	51 (15.6)	7 (11.7)	0.428
Complete transposition of great arteries	36 (11.0)	6 (10.0)	0.812
Congenitally corrected transposition of great arteries	46 (14.1)	4 (6.7)	0.115
**Dominant ventricle**, ***n*** **(%)**			
Left	59 (18.1)	9 (15.0)	0.563
Right	40 (12.3)	16 (26.7)	0.004
Undetermined	21 (6.4)	5 (8.3)	0.867
**Ventricle morphology**, ***n*** **(%)**			
Intermediated	205 (62.9)	37 (61.7)	0.858
Left	56 (17.2)	13 (21.7)	0.404
Right	66 (20.2)	10 (16.7)	0.525
**Atrioventricular valve regurgitation**, ***n*** **(%)**			
Trivial to mild	103 (31.6)	13 (21.7)	0.123
Moderate to severe	25 (7.7)	5 (8.3)	0.401
Ejection fraction (%)	63.1 ± 6.3	61.8 ± 5.0	0.155
**Cardiac catheterization at pre-TCPC**			
Mean pulmonary pressure (mm Hg)	12.5 ± 4.1	13.1 ± 3.2	0.352
Ventricular end-diastolic pressure (mm Hg)	9.7 ± 5.3	9.4 ± 4.7	0.851
Nakata index (mm^2^/m^2^)	278.3 (184.8, 361.6)	325.5 (183.5, 418.8)	0.536
McGoon ratio	2.1 (1.8, 2.5)	2.1 (1.7, 2.6)	0.875
Mean aortic pressure (mm Hg)	77.5 (68.5, 90.0)	74.0 (64.0, 81.0)	0.076
CVP (mm Hg)	12.0 (9.0, 13.3)	12.0 (10.0, 15.0)	0.990
**Pre-operative palliative operations**, ***n*** **(%)**			
Bidirectional Glenn	221 (67.8)	40 (66.7)	0.864
Pulmonary artery banding	9 (2.8)	0 (0)	0.403
Systemic pulmonary artery shunt	7 (2.1)	2 (3.3)	0.925
Interval from bidirectional Glenn to TCPC (years)	3.0 (2.0, 4.0)	3.0 (2.3, 4.0)	0.521
**Intraoperative variables**			
Cardiopulmonary bypass time (min)	103.0 (81.5, 140.8)	114.0 (89.0, 151.0)	0.101
Aortic cross clamping, *n* (%)	91 (28.1)	18 (30.5)	0.705
Cross-clamping time (min)	66.0 (50.0, 90.0)	71.5 (47.3, 99.5)	0.851
Fenestration at TCPC, *n* (%)	91 (27.9)	24 (40.0)	0.060
Extracardiac tunnel (vs. lateral tunnel)	318 (8)	59 (1)	0.591
**Concomitant procedure**, ***n*** **(%)**			
Atrioventricular valve repair	35 (10.7)	3 (5.0)	0.170
Pulmonary artery repair	22 (6.7)	5 (8.33)	0.867
**Post-operative variables**			
Peak CVP during the POD 0 (mm Hg)	12.0 (11.0, 14.0)	13.0 (11.8, 15.0)	0.046
Mechanical ventilation time (h)	21.9 (17.1, 29.0)	22.9 (17.6, 55.8)	0.047
ICU duration (days)	2.4(1.0, 4.6)	3.7 (1.7, 10.0)	0.000
Length of post-operative hospital stay (days)	27.0 (18.9, 39.1)	38.8 (27.8, 52.3)	0.000
Chest drainage duration (days)	12.0 (7.0, 21.0)	24.5 (15.8, 41.3)	0.000
Chest drainage volume (ml/day)	155.2 (104.3, 238.5)	229.8 (151.8, 345.0)	0.000

*Values are presented as n (%), mean ± standard deviation (SD), or median [interquartile range (IQR)]. CVP, central venous pressure; ICU, intensive care unit; POD, post-operative day; TCPC, total cavopulmonary connection*.

Chylothorax occurred in 60 patients (15.5%). The chylothorax group had a significantly higher proportion of age at surgery ≤ 3.5 years (23.3 vs. 13.5%, *p* = 0.044) and a higher percentage of right dominant ventricle (26.7 vs. 12.3%, *p* = 0.004) than the non-chylothorax group. Patients with chylothorax had significantly longer duration (24.5 vs. 12.0 days, *p* = 0.000) and higher volume (median, 229.8 vs. 155.2 ml/kg, *p* = 0.000) of post-operative chest tube drainage than those without chylothorax. The peak CVP during the POD 0 was higher in the chylothorax group than the non-chylothorax group (median, 13.0 vs. 12.0 mm Hg, *p* = 0.046). The duration of mechanical ventilation [22.9 (17.6, 55.8) vs. 21.9 (17.1, 29.0) h, *p* = 0.047], ICU stay [3.7 (1.7, 10.0) vs. 2.4 (1.0, 4.6) days, *p* = 0.000], and post-operative hospital stay [38.8 (27.8, 52.3) vs. 27.0 (18.9, 39.1) days, *p* = 0.000] were significantly longer in patients with chylothorax when compared with those without chylothorax. Comparisons of the two groups showed no differences in terms of pre-operative cardiac catheterization parameters, the degree of atrioventricular valve regurgitation, and intraoperative variables (*p* > 0.05).

### Risk Factors for Chylothorax

As shown in Table 2, the univariable analysis showed that parameters of weight, age younger than 3.5 years, right dominant ventricle, fenestration at TCPC, and peak CVP during POD 0 were significantly different between groups with and without chylothorax. After adjusting for potential confounders in multivariate analysis, having a right dominant ventricle [odds ratio (OR) = 2.711, 95% confidence interval (CI) = 1.285–5.721, *p* = 0.009] and a higher peak CVP on POD 0 (OR = 1.116, 95% CI = 1.011–1.233, *p* = 0.030) were the risk factors for the development of chylothorax after TCPC operation.

**Table 2:** Univariable and multivariable analyses of risk factors for chylothorax.

**Variables**	**Univariate analysis**			**Multivariate analysis**		
	**OR**	**95% CI**	***p*-Value**	**OR**	**95% CI**	***p*-Value**
Age ≤ 3.5 years	1.951	0.991–3.840	0.050	1.514	0.683–3.353	0.307
Weight (per 5 kg)	0.933	0.875–0.995	0.035	0.789	0.556–1.120	0.184
Right dominant single ventricle	2.600	1.343–5.035	0.005	2.711	1.285–5.721	0.009
Fenestration at TCPC	1.722	0.973–3.045	0.062	1.577	0.828–3.002	0.166
CPB time (per 10 min)	1.013	0.980–1.047	0.432			
Aortic cross clamping	1.124	0.614–2.058	0.705			
Peak CVP during the POD 0 (mm Hg)	1.106	1.006–1.217	0.038	1.116	1.011–1.233	0.030

*CI, confidence interval; CPB, cardiopulmonary bypass; CVP, central venous pressure; OR, odds ratio; POD, post-operative day; TCPC, total cavopulmonary connection*.

### Early- and Long-Term Outcomes

Early-term outcomes after TCPC operation between the two groups are shown in Table 3. One patient died 37 days after surgery because of multiple organ dysfunction syndrome. There were 12 late deaths in our cohort (seven in the chylothorax group and five in the non-chylothorax group) over the median follow-up period of 4.0 (2.0, 6.8) years. The survival rate of patients in the entire cohort at 5 and 10 years after the TCPC operation was 97.5 and 90.5%, respectively.

**Table 3:** Early-term outcomes after TCPC operation between patients with and without chylothorax.

**Variables**	**Non-chylothorax group**	**Chylothorax group**	***p*-Value**
	**(*n* = 326)**	**(*n* = 60)**	
Mechanical ventilation time (h)	21.8 (17.0, 28.4)	22.9 (17.3, 55.9)	0.047
ICU duration >5 days, *n* (%)	68 (21.2)	30 (42.9)	0.000
Post-operative hospital stay >41 days, *n* (%)	64 (20.0)	32 (45.7)	0.000
**Post-operative complications**, ***n*** **(%)**			
Death	1 (0.3)	0 (0.0)	1.000
ECMO	2 (0.6)	1 (1.7)	0.298
Infection	30 (9.2)	14 (23.3)	0.002
Prolonged pleural effusion (>14 days)	120 (36.8)	49 (81.7)	0.000
Ascites	21 (6.4)	12 (20.0)	0.001
Supraventricular tachycardia	4 (1.2)	3 (5.0)	0.137
Ventricular fibrillation	2 (0.6)	0 (0.0)	1.000
Diaphragmatic paralysis	4 (1.2)	4 (6.7)	0.026
Delayed sternal closure	5 (1.5)	2 (3.3)	0.665

*Values are presented as median [interquartile range (IQR)] or n (%). ECMO, extracorporeal membrane oxygenation; ICU, intensive care unit*.

**Figure 2:**
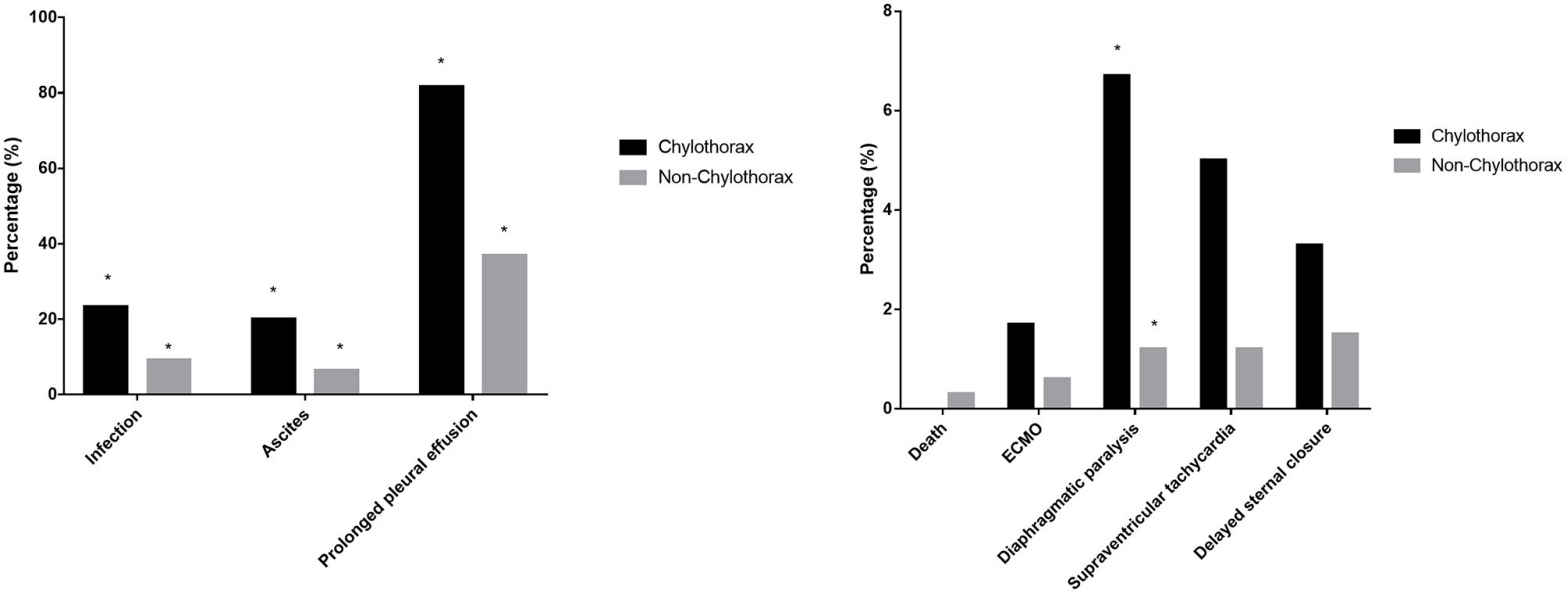
Early-term complications after TCPC operation between patients with and without chylothorax. *p < 0.05 between the two groups.

**Figure 3:**
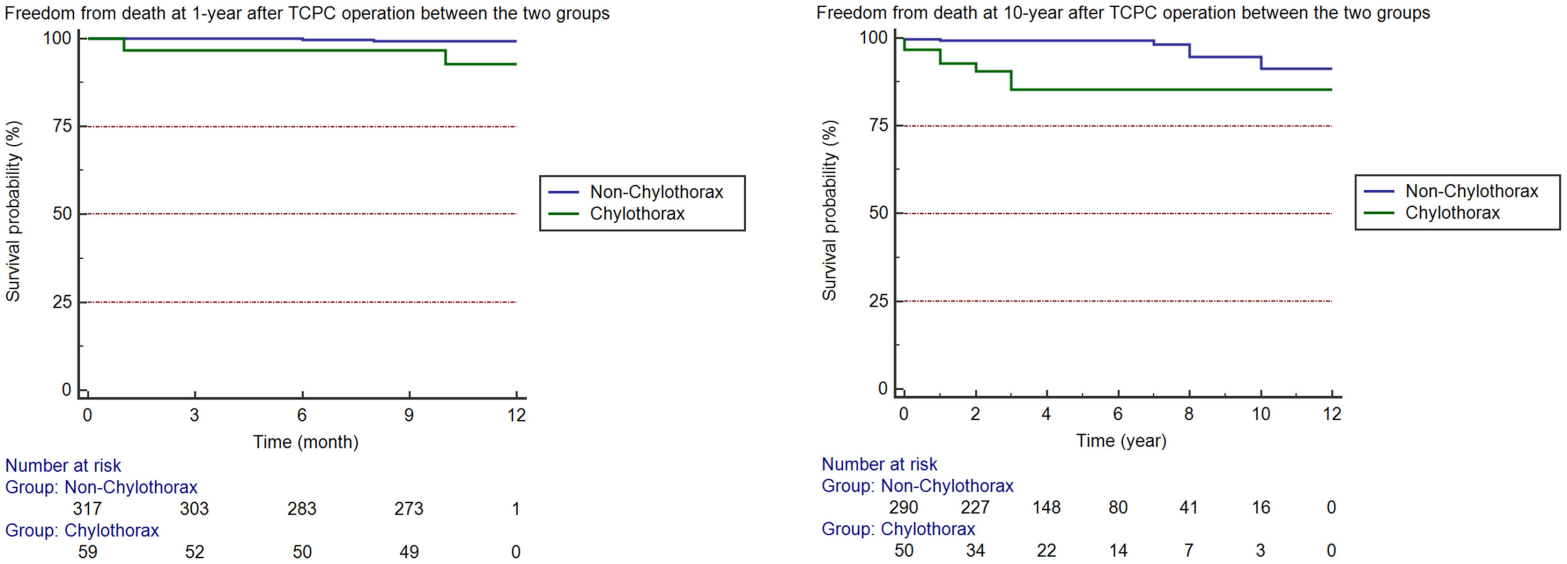
Freedom from death at 1 and 10 years after TCPC operation between the two groups.

In-hospital mortality, the incidence of ECMO support, arrhythmia, and delayed sternal closure were comparable between two groups. Patients with chylothorax had a higher incidence of infection (*p* = 0.002), ascites (*p* = 0.001), prolonged pleural effusion (*p* = 0.000), and diaphragmatic paralysis (*p* = 0.026). Besides, the prevalence of prolonged ICU stay (*p* = 0.000) and prolonged post-operative hospital stay (*p* = 0.000) in chylothorax group were significantly higher than non-chylothorax group (Figure 2). We also investigated the long-term survival in patients after TCPC surgery. The median follow-up duration was 4.0 (2.0, 6.8) years. The chylothorax group had significantly lower freedom from death at 1 year (92.4 vs. 99.3%, *p* < 0.001) and 10 years (84.6 vs. 91.6%, *p* < 0.001) than the non-chylothorax group, respectively (Figure 3).

## Discussion

The overall incidence of chylothorax after TCPC operation was 15.5% in our study, which was significantly lower than the 24% reported by previous studies (6, 7). Our results confirmed that patients with chylothorax after TCPC have an increased rate of early-term adverse events. This result is consistent with the finding of Schumacher et al. (9), who showed that chylothorax after Fontan was associated with the occurrence of plastic bronchitis and protein-losing enteropathy. The most important finding of this study is that patients with chylothorax after TCPC had not only higher incidence of early-term complications, but also lower survival rates at 1 and 10 years after hospital discharge, respectively. Risk factors identified in this study for the development of post-TCPC chylothorax included the right ventricle dominance and high CVP on POD 0.

Total cavopulmonary connection procedure involves redirecting the systemic venous return to the pulmonary arteries bypassing the right ventricle; this special circulation, although effective for many patients and with excellent early survival, inevitably exposes the systemic venous and lymphatic system to substantially higher pressure, which is associated with complications in the later stages (10). As the systemic venous pressure needs to be elevated to maintain forward flow into the pulmonary circulation, the raised CVP after TCPC results in elevated mean capillary hydrostatic pressure at the venular end and finally decreased reabsorption of the interstitial fluid. The imbalance of capillary filtration equilibrium leads to the development of pleural and pericardial effusions in the early period after TCPC operation. In addition to increased lymph production, the increase in CVP also makes it more difficult for chyle in the thoracic duct to empty into the great veins, which contributes to significant lymphatic congestion and dilatation of the thoracic duct (11). Ghosh et al. (12) applied pre-operative T2-weighted magnetic resonance imaging (MRI) sequences to assess for lymphatic perfusion abnormality and found that the T2 imaging has the potential to improve pre-operative risk stratification and enable identification of patients with high-grade lymphatic abnormalities. The application of this technique to evaluate the function and change of the thoracic duct may provide perception into early identification and treatment for chylothorax after TCPC.

Lo Rito et al. (7) did not find any risk factors for chylothorax after the Fontan operation whereas Soquet et al. (6) reported that the right ventricle dominance was significantly associated with the occurrence of chylothorax after cavopulmonary connection. Consistent with Soquet et al., our study found that the right ventricle dominance was an independent risk factor for chylothorax after TCPC operation. It was suggested that the increased ventricular diastolic pressure in patients with dominant right ventricles would be expected to elevate the CVP and subsequently lymphatic pressure. The mechanism by which a right ventricular dominance increased the risk of chylothorax after Fontan operation is complex and unclear. We hypothesized that the specific pathophysiological factors of right ventricular dominance may be related to post-operative chylothorax. The Fontan circulation has been shown to increase the demand on the single ventricle secondary to a mismatch between contractility and afterload with the systemic right ventricle seemingly more negatively affected. Staged surgical palliation before TCPC operation has become a standard practice, which allows significant unloading of the single ventricular volume and results in decreased end-diastolic volume (13, 14). Although with these refinements, morphological right ventricle anatomy has been identified as an independent predictor of heart failure death and complications after Fontan surgery (15, 16). The other notable finding of our study was that higher CVP on POD 0 was associated with the occurrence of chylothorax. This finding further indicated that the inevitably increased CVP after Fontan is a challenge for the lymphatic circulation. The anatomic lymphatic changes using non-contrast MRI were investigated, and it was found that patients with a Fontan circulation have an impaired lymphatic capacity and morphologically changed thoracic duct (17). The capillary filtration of fluid is increased because of elevated CVP, and more fluid from the lymphatic vessels needs to be removed to maintain normal tissue fluid balance.

Our data showed no statistically different incidence of fenestration between the chylothorax group and non-chylothorax group, but the creation of fenestration contributes to decreased duration and volume of the chest tube drainage in our study; its ability to reduce chylothorax by reducing CVP as expected remains unclear. Airan et al. (18) also found that patients with a fenestrated Fontan procedure had a lower amount and duration of pleural effusion. However, a recent prospective randomized study performed by the same institute found that a fenestration in the extracardiac Fontan circuit was not associated with a reduction in the amount and duration of pleural effusion compared with a non-fenestrated Fontan (19). Whether fenestration during operation improves the pleural effusion and reduces the risk of lymphatic circulation disorder needs further study.

Therapy strategies are aimed at decreasing lymph flow to allow the thoracic duct to heal, at the same time preventing and treating complications associated with the persistent loss of chyle (20). Most patients with chylothorax after cardiac surgery respond to aggressive medical treatment without the requirement of surgical treatment. The medical treatment for chylothorax in our study consists of the following: dietary modification of fat-free formula, total parenteral nutrition, and octreotide or stilamin. Besides, albumin supplementation was routinely applied to increase the colloid osmotic pressure to reduce interstitial edema. Only one patient in our cohort was transferred to another hospital for the refractory chylothorax, and none of the others required thoracic duct ligation as patients improved over time under the therapy of traditional medical management.

The impact of chylothorax is considerable as it is associated with increased morbidity and mortality after TCPC operation. Mery et al. (2) analyzed a large multi-institution database of children undergoing cardiac surgery; they found that chylothorax was associated with in-hospital mortality, the length of hospital stay, and increased hospitalization cost. Our analysis showed that the occurrence of chylothorax not only significantly prolonged the mechanical ventilation time, the ICU duration, and the post-operative hospital stay, but was also associated with a higher risk of late mortality after hospital discharge. These results may be related to significant loss of lipids, protein, lymphocytes, and electrolytes, which can induce nutritional and metabolic complications (21). Lo Rito et al. (7) reported a similar trend that patients who experience chylothorax in the acute post-operative period are at a higher risk of late death and adverse events.

One of the major limitations to our study was the single-center retrospective design; the patient selection criteria can hardly be completely random, and the inherent bias associated with a retrospective study thus exists. In addition, the occurrence of chylothorax after palliative operation was not recorded, which might also be the potential risk factor of subsequent chylothorax after TCPC. Finally, as we did not have the hemodynamic data from cardiac catheterization before TCPC operation, such as pulmonary resistance, the evaluation of pre-operative pulmonary circulation was incompletely.

In conclusion, the incidence of chylothorax in patients undergoing TCPC is lower than previously reported, but is associated with poor early- and long-term survival. Chylothorax following TCPC operation could be a sign of poorer long-term outcomes. Having a right dominant ventricle and a higher peak CVP on POD 0 are significant risk factors for chylothorax after TCPC. Identifying the potential risk factors for chylothorax in Fontan patients may help to perform an early risk stratification for these vulnerable patients and improve their outcomes. Further exploration of the function and structure of the lymphatic system in TCPC patients during the perioperative period may help to an in-depth understanding of the prevention and treatment of post-operative chylothorax.

## Data Availability Statement

The original contributions presented in the study are included in the article/supplementary material, further inquiries can be directed to the corresponding author/s.

## Ethics Statement

The studies involving human participants were reviewed and approved by the Ethics Committee of Fuwai Hospital. Written informed consent from the participants' legal guardian/next of kin was not required to participate in this study in accordance with the national legislation and the institutional requirements.

## Author Contributions

LB: study design, statistical analysis, and manuscript preparation. JL: study design and manuscript review. ZF and JZ: clinical management. SG: statistical analysis. YT and YJ: data acquisition. PZ and PG: provided critical revision. YL: literature search. All authors contributed to the article and approved the submitted version.

## Funding

This study was funded by the National Natural Science Fund (grant number 81670375).

## Conflict of Interest

The authors declare that the research was conducted in the absence of any commercial or financial relationships that could be construed as a potential conflict of interest.

## Publisher's Note

All claims expressed in this article are solely those of the authors and do not necessarily represent those of their affiliated organizations, or those of the publisher, the editors and the reviewers. Any product that may be evaluated in this article, or claim that may be made by its manufacturer, is not guaranteed or endorsed by the publisher.
